# Spurious violation of the Stokes–Einstein–Debye relation in supercooled water

**DOI:** 10.1038/s41598-019-44517-4

**Published:** 2019-05-31

**Authors:** Takeshi Kawasaki, Kang Kim

**Affiliations:** 1 Department of Physics, Nagoya University, Nagoya, 464-8602 Japan; 2 Division of Chemical Engineering, Graduate School of Engineering Science, Osaka University, Toyonaka, Osaka 560-8531 Japan; 3 Institute for Molecular Science, Okazaki, Aichi 444-8585 Japan

**Keywords:** Chemical physics, Chemical physics

## Abstract

The theories of Brownian motion, the Debye rotational diffusion model, and hydrodynamics together provide us with the Stokes–Einstein–Debye (SED) relation between the rotational relaxation time of the ${\boldsymbol{\ell }}$-th degree Legendre polynomials ${{\boldsymbol{\tau }}}_{{\boldsymbol{\ell }}}$, and viscosity divided by temperature, *η*/*T*. Experiments on supercooled liquids are frequently performed to measure the SED relations, ${{\boldsymbol{\tau }}}_{{\boldsymbol{\ell }}}$*k*_B_*T*/*η* and *D*_t_${{\boldsymbol{\tau }}}_{{\boldsymbol{\ell }}}$, where *D*_t_ is the translational diffusion constant. However, the SED relations break down, and its molecular origin remains elusive. Here, we assess the validity of the SED relations in TIP4P/2005 supercooled water using molecular dynamics simulations. Specifically, we demonstrate that the higher-order ${{\boldsymbol{\tau }}}_{{\boldsymbol{\ell }}}$ values exhibit a temperature dependence similar to that of *η*/*T*, whereas the lowest-order ${{\boldsymbol{\tau }}}_{{\boldsymbol{\ell }}}$ values are decoupled with *η*/*T*, but are coupled with the translational diffusion constant *D*_t_. We reveal that the SED relations are so spurious that they significantly depend on the degree of Legendre polynomials.

## Introduction

Characterization of the translational and rotational motions of molecules in liquid states is of great significance^1–3^. For this purpose, various transport properties, such as shear viscosity, translational diffusion constant, and rotational relaxation time have been measured both experimentally, and through molecular dynamics (MD) simulations. These properties also play crucial roles in the understanding of the detailed mechanism of hydrogen-bond network dynamics in liquid water^4–11^.

The Stokes–Einstein (SE) relation is one of the important characteristics of the translational diffusion constant, *D*_t_, in many liquid state systems, ${D}_{{\rm{t}}}={k}_{{\rm{B}}}T$/$(6\pi \eta R)$, where *k*_B_, *T*, *η* represent the Boltzmann constant, the temperature, and the shear viscosity, respectively. This SE relation is derived originally from the theories of hydrodynamics and Brownian motion, where a rigid spherical particle with a radius *R* is assumed to be perfectly suspended in a Stokes flow of a constant shear viscosity *η* under the stick boundary condition^12^. Thus, *R* is conventionally regarded as the effective hydrodynamic radius of the molecule when applying the SE relation to molecular liquids^13^.

Analogous to translational motion, the rotational Brownian motion leads to another SE relation between the rotational diffusion constant, *D*_r_, and *η* as ${D}_{{\rm{r}}}={k}_{{\rm{B}}}T$/$(8\pi \eta {R}^{3})$. Based on the Debye model, *D*_r_ can also be determined by solving the rotational diffusion equation for the reorientation of the molecular dipole as ${D}_{{\rm{r}}}=1$/$[{\tau }_{\ell }\ell (\ell +1)]$, where ${\tau }_{\ell }$ is the rotational relaxation time of the $\ell$-th order Legendre polynomials^14^. ${\tau }_{1}$ and ${\tau }_{2}$ are the most-commonly investigated; they are characterized by dielectric relaxation and NMR spectroscopies, respectively. Note that a deviation from ${\tau }_{1}$/${\tau }_{2}=3$ has been reported in supercooled molecular liquids, which is regarded as a sign of the breakdown of the Debye model^15–17^. Those two equations result in the Stokes–Einstein–Debye (SED) relation,

1 $$\frac{{\tau }_{\ell }{k}_{{\rm{B}}}T}{\eta }=\frac{8\pi {R}^{3}}{\ell (\ell +1)}.$$

The SED relation can also be expressed as

2 $${D}_{{\rm{t}}}{\tau }_{\ell }=\frac{4{R}^{2}}{3\ell (\ell +1)},$$

by combining further with the SE relation between *D*_t_ and *η*/*T*. This SED relation is proportional to the quotient *D*_t_/*D*_r_, which accounts for the coupling between the translational and rotational diffusion dynamics at any temperature.

The violation of the SE relation between *D*_t_ and *η* has been intensively observed in various glass-forming liquids, such as *o*-terphenyl^18–23^. In particular, the quantity ${D}_{{\rm{t}}}\eta$/*T* increases towards the glass transition temperature, but exhibits a constant value at high temperatures. These experiments indicate that the translational diffusion occurs in a more enhanced manner than estimations using shear viscosity. Many theoretical efforts have therefore been devoted to explaining the violation of the SE relation in glass-forming liquids^24–32^. MD simulations have also been variously performed to address their molecular mechanisms^33–39^. It is commonly argued that the violation of SE relation is a sign of spatially heterogeneous dynamics and of the non-Gaussian property of the particle displacement distribution^22,40^.

The validity of the SED relation is still highly controversial, because there are three possible candidates, ${\tau }_{\ell }T$/*η*, ${D}_{{\rm{t}}}{\tau }_{\ell }$, and *D*_t_/*D*_r_, that need to be quantified. Recently, the *D*_r_ of supercooled molecular liquids has been calculated using MD simulations following the Einstein relation for rotational Brownian motions^41–43^. Experimental analogs have also been reported using optical spectroscopy in colloidal glasses^44,45^. In particular, it has been shown that the temperature dependences of ${D}_{{\rm{t}}}{\tau }_{2}$ and *D*_t_/*D*_r_ are completely different in *o*-terphenyl liquids^42^ and diatomic molecular liquids^43^; *D*_t_/*D*_r_ significantly decreases with decreasing temperature, indicating the translational-rotational decoupling. In contrast, ${D}_{{\rm{t}}}{\tau }_{2}$ exhibits the opposite temperature dependence, *i*.*e*., increases in ${\tau }_{2}$ exceed the time scale of the translational diffusion constant, 1/*D*_t_, as the temperature decreases. This discrepancy is thus attributed to the inconsistency between the two expressions, Eqs (1) and (2). However, the direct measurement of *R* is impractical for molecular liquids both in experiments and MD simulations. More practically, the breakdown of the Debye model, *i*.*e*., the $\ell$ dependence of ${\tau }_{\ell }$, prevents us from making a precise assessment of the SED relation, whichever one of three quantities is utilized.

For liquid water, it has been widely accepted that the validity of the Debye model for molecular reorientation is limited even in normal states, although that is frequently used when analyzing experimental data. Instead, various large-amplitude rotational jump models have been developed to give an accurate prediction of the rotational relaxation time ${\tau }_{2}$^7,8,11^. Particularly for supercooled water, the appropriate description for the violation of the SED relation becomes more complicated. Recent MD simulations have demonstrated that the translational and rotational dynamics become spatially heterogeneous upon cooling^46,47^. Furthermore, the violations of the SE and SED relations have been intensely characterized through both experiments and simulations^11,48–67^. In particular, the violation of the SED relation and the translational-rotational decoupling in supercooled water have been reported by calculating *D*_t_ and *D*_r_, while $\eta$ has not been calculated^49,52,57^, despite the fact that $\eta$ plays an essential role in the precise assessment of the SE relation^60–63^. The SED relation has been investigated by calculating the $\eta$ of SPC/E supercooled water, during which *D*_r_ was not calculated^62^. Under these conditions, the SED relation, particularly for the $\ell$ dependence of ${\tau }_{\ell }T$/*η*, has not yet been thoroughly investigated, while only one experimental data analysis has been conducted for ${\tau }_{2}T$/*η*^59^.

The purpose of this study is to shed light on the controversy regarding the violation of the SED relation, specifically through the numerical calculations of three quantities, ${\tau }_{\ell }T$/*η*, ${D}_{{\rm{t}}}{\tau }_{\ell }$, and *D*_t_/*D*_r_. In particular, we aim to demonstrate that the $\ell$ dependence of ${\tau }_{\ell }T$/*η* is an important factor in exploring the inherent translational-rotational dynamics in supercooled water.

## Results

**Figure 1:**
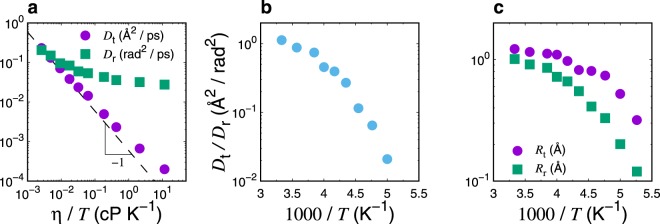
(**a**) Assessments of the translational and rotational SE relations, ${D}_{{\rm{t}}}\eta$/*T* and ${D}_{{\rm{r}}}\eta$/*T*, made by plotting the relationships between the translational diffusion constant *D*_t_ or the rotational diffusion constant *D*_r_ and the shear viscosity divided by the temperature *η*/*T*. The dashed line represents the linear relation ${D}_{{\rm{t}},{\rm{r}}}\propto \eta$/*T*, which represents the SE relation. Neither the translational nor the rotational SE relations are satisfied in supercooled region (*T* < 250 K). (**b**) Temperature dependence of the ratio of rotational and translational diffusion constants, *D*_r_/*D*_t_. As *T* decreases, this ratio increases, indicating the translational-rotational diffusion decoupling. (**c**) Temperature dependence of translational and rotational hydrodynamic radii, *R*_t_ and *R*. Both *R*_t_ and *R*_r_ decrease significantly upon cooling, accompanied with violation of SE relations. In particular, upon cooling, *R*_r_ decreases at a higher rate than that of *R*_t_ in response to decreasing temperature.

Here we examine the translational and rotational SE relations, ${D}_{{\rm{t}}}\propto \eta$/*T* and ${D}_{{\rm{r}}}\propto \eta$/*T*, respectively. We determined *D*_t_ and *D*_r_ from the long-time behaviors of the translational and rotational mean-square displacements, respectively (see Methods). We calculated *η* using the Green–Kubo formula for the shear stress correlation function, as detailed in a previous study^63^. Figure 1(a) shows both *D*_t_ and *D*_r_ as a function of *η*/*T*. Comparing these with the dashed line representing the linear relationship, we find that both the translational and the rotational SE relations are invalid in supercooled regimes, particularly at *T* < 250 K. Note that the rotational SE relation is violated to a greater extent than the translational SE relation. Figure 1(b) shows the ratio of the translational and rotational diffusion constants, *D*_t_/*D*_t_, as a function of the scaled inverse of temperature. The substantial decoupling displayed between the two diffusion constants indicates that the translational and rotational dynamics are decoupling, which is comparable with the previously reported results on ST2^49^, SPC/E^52^, and TIP4P/2005^57^ models. Furthermore, similar results are also demonstrated in *o*-terphenyl liquids^42^ and diatomic molecular liquids^41,43^ using MD simulations.

The observed decoupling of translational-rotational diffusion is directly related to the inconsistency regarding the effective hydrodynamic radius observed when using the SE relations. We quantified the hydrodynamic radius for the translational degree of freedom, R_t_ = k_B_*T*/$(6\pi \eta {D}_{{\rm{t}}})$, and the rotational counterpart, ${R}_{{\rm{r}}}={[{k}_{{\rm{B}}}T/(8\pi \eta {D}_{{\rm{r}}})]}^{1/3}$. Figure 1(c) shows the temperature dependences of *R*_t_ and *R*_r_. At *T* = 300 K, *R*_t_ and *R*_r_ are approximated by 1.2 Å and 1.0 Å, respectively. These values are slightly smaller than the van der Waals radius of the TIP4P/2005 model. As seen in Fig. 1(c), these two radii sharply decrease upon supercooling, accompanied by violation of the translational and rotational SE relations. Moreover, the difference between *R*_t_ and *R*_r_ increases with decreasing the temperature, implying that the translational and rotational diffusions are decoupling. The relevance of the decoupling *D*_t_/*D*_r_ will be discussed below.

**Figure 2:**
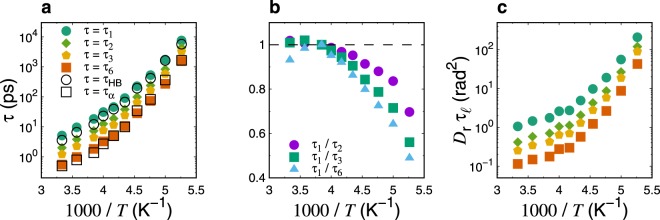
(**a**) Temperature dependence of $\ell$-th order rotational relaxation times ${\tau }_{\ell }$ for $\ell =1$, 2, 3, and 6. The temperature dependences of hydrogen-bond lifetime, ${\tau }_{{\rm{HB}}}$, and *α*-relaxation time, ${\tau }_{\alpha }$, are included for comparison. (**b**) Temperature dependence of ratio ${\tau }_{1}$/${\tau }_{2}$, ${\tau }_{1}$/${\tau }_{3}$, and ${\tau }_{1}$/${\tau }_{6}$. Each quantity is scaled by the value at *T* = 260 K. (**c**) Assessments of the Debye model, made by plotting the temperature dependence of ${D}_{{\rm{r}}}{\tau }_{\ell }$ for $\ell =1$, 2, 3, and 6.

Next, we investigate ${\tau }_{\ell }$ as determined from the $\ell$-th order Legendre polynomials, and explore its relationship with *D*_r_. Figure 2(a) shows ${\tau }_{\ell }$ (for $\ell =1$, 2, 3, and 6) as a function of the scaled inverse of the temperature. ${\tau }_{\ell }$ increases for all $\ell$ values as the temperature decreases. Interestingly, we observe that ${\tau }_{\ell }$ values with higher-order degrees exhibit stronger temperature dependence than those of the lowest order. In other words, the ratios ${\tau }_{1}$/${\tau }_{2}$, ${\tau }_{1}$/${\tau }_{3}$, and ${\tau }_{1}$/${\tau }_{6}$ notably decrease as the temperature decreases (see Fig. 2(b)). A similar result was found using MD simulations of the SPC/E supercooled water^62^. As evident in Fig. 2(b), ${D}_{{\rm{r}}}{\tau }_{\ell }$ exhibits strong temperature dependence, indicating the breakdown of the Debye model. The observed deviation increases for higher-order $\ell$ values with decreasing temperatures. The breakdown of the Debye model and the inconsistency between *R*_t_ and *R*_r_ in supercooled states suggest that the SED relations, ${\tau }_{\ell }T$/*η*, ${D}_{{\rm{t}}}{\tau }_{\ell }$, and *D*_t_/*D*_r_, are likely spurious quantities. More precisely, these quantities cannot represent real translational and rotational dynamics in supercooled water, regardless of whether they exhibit anomalous deviations from values at high temperatures. We below demonstrate ambiguities of the SED relations, of which results markedly depend on the order $\ell$.

**Figure 3:**
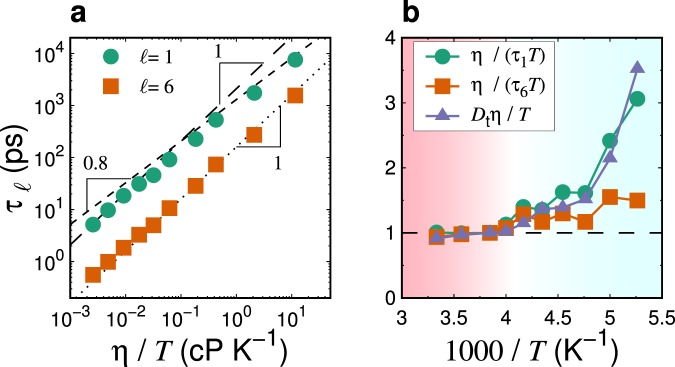
(**a**) Assessments of the SED relation, ${\tau }_{\ell }T$/*η*, made by plotting the relationships between rotational relaxation time ${\tau }_{\ell }$ for $\ell =1$ and $\ell =6$, and the shear viscosity divided by the temperature, *η*/*T*. Both the dotted line and the long-dashed line represent the SED relation, ${\tau }_{\ell }\propto \eta$/*T*. The short-dashed line represents ${\tau }_{\ell }\propto {(\eta /T)}^{0.8}$. (**b**) Assessments of the SED relation, ${\tau }_{\ell }T$/*η*, made by plotting the temperature dependence of *η*/$({\tau }_{\ell }T)$ for $\ell =1$ and $\ell =6$. The violation of the SE relation, ${D}_{{\rm{t}}}\eta$/*T*, is also plotted for comparison. Each quantity is scaled by the value at *T* = 260 K. The SED ratio *η*/$({\tau }_{1}T)$ exhibits a temperature dependence similar to the violation of the SE relation, ${D}_{{\rm{t}}}\eta$/*T*, whereas the plot of *η*/$({\tau }_{6}T)$ resembles the SED relation, Eq. (1). The background color (white region) indicates the onset temperature of the SE violation.

Here, we address the SED relation ${\tau }_{\ell }T$/*η*, which is the counterpart to recent experimental data^59^. Figure 3(a) shows the relationship between *η*/*T* and ${\tau }_{\ell }$ for $\ell =1$ and $\ell =6$. Note that the results of $\ell =2$ and $\ell =3$ are omitted from the plot to improve its clarity. As observed in Fig. 3(a), ${\tau }_{1}$ deviates from the value predicted by the SED relation, particularly at lower temperatures (*T* < 250 K), instead exhibiting the fractional form ${\tau }_{1}\propto {(\eta /T)}^{-0.8}$. In contrast, ${\tau }_{\ell }$ with at higher-order $\ell =6$ follows the SED relation, ${\tau }_{6}\propto \eta$/*T*. Figure 3(b) shows the temperature dependence of $\eta$/$({\tau }_{\ell }T)$ (for $\ell =1$ and $\ell =6$), in comparison with that of the translational SE relation, ${D}_{{\rm{t}}}\eta$/*T*. We observe that the temperature dependence of $\eta$/$({\tau }_{1}T)$ is analogous to that of ${D}_{{\rm{t}}}\eta$/*T*, suggesting the violation of the SE relation, whereas $\eta$/$({\tau }_{6}T)$ exhibits a weaker temperature dependence. We previously demonstrated the relationship ${\tau }_{\alpha }\propto \eta$/*T* in TIP4P/2005 supercooled water^63^. Here, ${\tau }_{\alpha }$ denotes the *α*-relaxation time that was determined from the incoherent intermediate scattering function *F*_*s*_(*k*, *t*). The wave-number, *k*, was chosen as *k* = 3.0 Å^−1^, which corresponds to the main peak of the static structure factor of oxygen, *S*_OO_(*k*). This implies that ${D}_{{\rm{t}}}{\tau }_{\alpha }$ is a good proxy for the translational SE relation ${D}_{{\rm{t}}}\eta$/*T*. Similar results have also been reported for other supercooled liquid systems^34,38,39,68^. Accordingly, the temperature dependence of ${\tau }_{\ell }$ with higher-order $\ell$ resembles the coupling with that of ${\tau }_{\alpha }$ (see Fig. 2(a)). On the other hand, the deviation of *η*/$({\tau }_{1}T)$ from this value at high temperatures superimposes the violation of the translational SE relation, ${D}_{{\rm{t}}}\eta$/*T* or ${D}_{{\rm{t}}}{\tau }_{\alpha }$.

**Figure 4:**
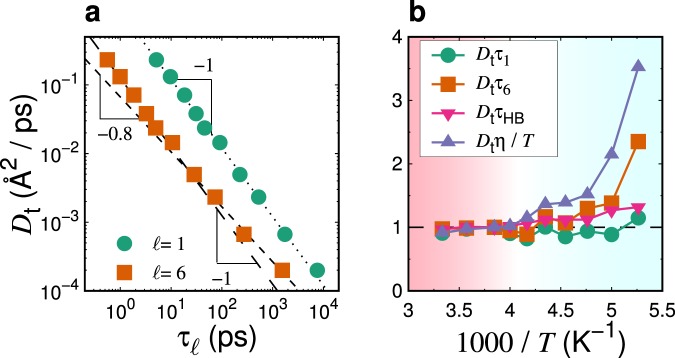
(**a**) Assessments of the SED relation, ${D}_{{\rm{t}}}{\tau }_{\ell }$, made by plotting the relationship between the translational diffusion constant, *D*_t_, and the rotational relaxation time, ${\tau }_{\ell }$, for $\ell =1$ and $\ell =6$. Both the dotted line and the long-dashed line represent the SED relation, ${D}_{{\rm{t}}}\propto {\tau }_{\ell }^{-1}$. The short-dashed line represents ${D}_{{\rm{t}}}\propto {\tau }_{\ell }^{-0.8}$. (**b**) Assessments of the SED relation, ${D}_{{\rm{t}}}{\tau }_{\ell }$, made by plotting the temperature dependence of the SED ratios ${D}_{{\rm{t}}}{\tau }_{\ell }$. The violation of SE relation, *D*t$\eta$/*T*, is also plotted for comparison. ${D}_{{\rm{t}}}{\tau }_{{\rm{HB}}}$, with hydrogen-bond lifetime ${\tau }_{{\rm{HB}}}$, is also shown. Each quantity is scaled by the value at *T* = 260 K. Note that ${D}_{{\rm{t}}}{\tau }_{{\rm{HB}}}$ shows the preservation of the SE relation^63^. The SED ratio *η*/$({\tau }_{1}T)$ exhibits a temperature dependence similar to the preservation of the SE relation, ${D}_{{\rm{t}}}{\tau }_{{\rm{HB}}}$, whereas ${D}_{{\rm{t}}}{\tau }_{6}$ bears a certain resemblance to the violation of the SE relation, ${D}_{{\rm{t}}}\eta$/*T*. The background color (white region) indicates of the onset temperature of the SE violation.

We next examine the second SED relation, ${D}_{{\rm{t}}}{\tau }_{\ell }$. Figure 4(a and b) display the relationship between *D*_t_ and ${\tau }_{\ell }$ and the temperature dependence of ${D}_{{\rm{t}}}{\tau }_{\ell }$, respectively. As ${\tau }_{6}$ can serve as a proxy of *η*/*T* as observed in Fig. 3, ${D}_{{\rm{t}}}{\tau }_{6}$ exhibits a comparable temperature dependence with the SE ratio ${D}_{{\rm{t}}}\eta$/*T* (see Fig. 4(b)). In contrast, ${\tau }_{1}$ exhibits a temperature dependence similar to that of *D*_t_. Furthermore, we show an alternative quantity, ${D}_{{\rm{t}}}{\tau }_{{\rm{HB}}}$, with the hydrogen-bond lifetime, ${\tau }_{{\rm{HB}}}$. Here, ${\tau }_{{\rm{HB}}}$ represents the time scale characterizing the irreversible hydrogen-bond breakage process, which is determined from the hydrogen-bond correlation function^69–73^. As demonstrated in a previous study and displayed in Fig. 4(b), the SE relation is preserved as ${D}_{{\rm{t}}}\propto {{\tau }_{{\rm{H}}{\rm{B}}}}^{-1}$ if the time scale ${\tau }_{\alpha }$ is replaced with ${\tau }_{{\rm{HB}}}$^63^. Similar observations have also been reported in binary soft-sphere supercooled liquids^74^ and silica-like network-forming supercooled liquids^68^. This SE preservation is understood by a possible “jump model”. As outlined in the ref.^63^, the frequency of the jump motion can be represented as $f\sim 1$/${\tau }_{{\rm{HB}}}$ at investigated temeperatues. Correspondingly, the translational diffusion constant is modeled as ${D}_{{\rm{t}}}\sim {\ell }_{{\rm{jump}}}^{2}\,f\sim {\ell }_{{\rm{jump}}}^{2}$/${\tau }_{{\rm{HB}}}$, where ${\ell }_{{\rm{jump}}}$ denotes a typical jump length (~Å). Therefore, ${D}_{{\rm{t}}}{\tau }_{{\rm{HB}}}$ becomes constant. This SE preservation indicates that irreversible hydrogen-bond breakages destroy the local tetrahedral structures, and lead to the translational and rotational molecular jumps with high mobility. It also implies the the coupling between ${\tau }_{1}$ and ${\tau }_{{\rm{HB}}}$, which is demonstrated in Fig. 4.

**Figure 5:**
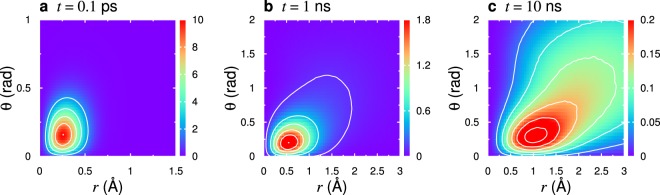
Joint probability distribution functions (generalized van Hove correlation function) $4\pi {r}^{2}{G}_{s}(r,\theta ;t)$ for *t* = 0.1 ns, 1 ns, and 10 ns at *T* = 190 K. The value of the color bar is normalized by Å^2^. Remarkable positive correlations between translational displacement $|{\rm{\Delta }}{\overrightarrow{r}}_{j}(t)|$ and rotational angle Δ${\theta }_{j}(t)$ are observed, particularly for large *r* and *θ* values. This indicates that a large translational motion correlates with a large rotational motion of molecule.

To elucidate the molecular mechanism of the demonstrated relationship between *D*_t_ and ${\tau }_{\ell }$, we analyze the generalized van Hove self correlation function, *i*.*e*., the joint probability distribution function for the translational displacement and the rotational angle of the molecule, ${G}_{s}(\overrightarrow{r},\theta ;t)=(1/N)\,{\sum }_{j=1}^{N}\,\langle \delta (\overrightarrow{r}-{\rm{\Delta }}{\overrightarrow{r}}_{j}(t))\delta (\theta -{\rm{\Delta }}{\theta }_{j}(t))\rangle$^75^. Here, Δ${\overrightarrow{r}}_{j}(t)$ and Δ${\theta }_{j}(t)$ are the translational displacement vector of oxygen and the rotational angle of the dipole moment of a molecule *j* during a time *t*, respectively. Figure 5 shows the contour maps of $4\pi {r}^{2}{G}_{s}(r,\theta ;t)$ with $r=|\overrightarrow{r}|$ for $t=0.1\,{\rm{ps}}$, 1 ns, and 10 ns at *T* = 190 K. For the shorter time interval, *t* = 0.1 ps, the distribution is stretched towards the rotational angle direction, *θ*, which is caused by the libration motion of the molecule. This observation corresponds to the oscillations of ${C}_{\ell }(t)$ (see Supplementary Fig. S2). At longer time scales, however, ${G}_{s}(r,\theta ;t)$ shows the coupling between the translational displacement and the rotational angle, which is consistent with the previously reported results of ref.^75^. Furthermore, the broad ridge separated from the main peak denotes the non-Gaussianity of ${G}_{s}(r,\theta ;t)$. A tagged molecule is trapped by a cage composed of neighbor molecules for longer times in supercooled regime. The rotational relaxation time ${\tau }_{1}$, of which the characteristic angle is *π*/2 rad, is governed by this large rotational mobility. The single molecule eventually begins diffusion by escaping from the cage, utilizing large translational and rotational mobilities. Thus, ${\tau }_{1}$ is regarded as the time scale coupled with *D*_t_. In addition, the time scale of ${\tau }_{1}$ is similar to the hydrogen-bond lifetime ${\tau }_{{\rm{HB}}}$, as demonstrated in Fig. 2(a).

In contrast, the relaxation time ${\tau }_{6}$ corresponds to a molecular reorientation with an angle of 0.37 rad, which lies near to the dominant peak of ${G}_{s}(r,\theta ;t)$ at *t* = 10 ns (see Fig. 5(c)). The higher-order $\ell$ mostly highlights immobile molecules both for translational and rotational motions, which will contribute to the dynamical heterogeneities. To investigate this, we characterize the dynamic heterogeneity of translational and rotational motions using the four-point correlation functions, ${\chi }_{{\rm{t}},{\rm{r}}}(t)$ (see Methods and Supplementary Fig. S3(a)). We found that the time scale ${\tau }_{6}$ is akin to the peak time of ${\chi }_{{\rm{t}},{\rm{r}}}(t)$, which shows that its temperature dependence is similar to that of ${\tau }_{\alpha }$ (see Supplementary Fig. S3(b)). Consequently, the similar temperature dependences between ${\tau }_{6}$ and ${\tau }_{\alpha }$ are demonstrated in Fig. 2(a).

Finally, we discuss the strong decoupling behavior of the translational and rotational diffusion constants, as demonstrated in Fig. 1. As already pointed out in ref.^8^, the use of the rotational diffusion constant *D*_r_ needs particular care due to the limitation of the angular Brownian motion scnenario. Furthermore, it has been revealed that *D*_r_ is superfacial for describing the reorientational motion in supercooled molecular liquids^43^. The angular mean-square displacement $\langle {\rm{\Delta }}\varphi {(t)}^{2}\rangle$ is largely influenced by the accumulation of the libration motion, which has a time scale of 0.1 ps. Each molecule can rotate, despite being trapped by the cage, at this short time scale, as indicated in Fig. 5(a). Accordingly, the angular mean-square displacement exhibits a plateau, but its persistent time is much smaller than that of ${C}_{\ell }(t)$, particularly at lower temperatures (See Supplementary Figs S1 and S2). In contrast, the plateau of ${C}_{\ell }(t)$ after the time scale of libration motion indicates the occurrence of the cage effect (see Supplementary Fig. S2). These findings imply that the decoupling between *D*_t_ and *D*_r_ has no direct relevance to the real translational-rotational coupling, ${D}_{t}\propto {\tau }_{1}^{-1}$. This translational-rotational coupling scenario is in accord with the observasion in supercooled molecular liquids^43^.

## Discussion

In this paper, we report the numerical results of MD simulations of the relationship between the translational and rotational dynamics in TIP4P/2005 supercooled liquid water. Our contributions to the assessment of translational and rotational SE relations and the Debye model can be summarized as follows:

(i) Both translational and rotational SE relations, ${D}_{{\rm{t}}}\propto {(\eta /T)}^{-1}$ and ${D}_{{\rm{r}}}\propto {(\eta /T)}^{-1}$, are significantly violated in supercooled states. In particular, the rotational SE relation is violated stronger to a greater extent than that of translational SE relation. Correspondingly, the rotational hydrodynamic radius becomes significantly smaller than translational one with decreasing temperature.
(ii) We test the validity of the Debye model, ${D}_{{\rm{r}}}\propto {{\tau }_{\ell }}^{-1}$, for the orders $\ell =1$, 2, 3, and 6 of the Legendre polynomials, demonstrating that the rotational relaxation time ${\tau }_{\ell }$ is entirely inconsistent with the rotational diffusion constant *D*_r_.
(iii) Furthermore, we systematically examine the SED relations ${\tau }_{\ell }T$/*η* and ${D}_{{\rm{t}}}{\tau }_{\ell }$. We reveal that these SED relations strongly depend on the order of $\ell$, leading to the following spurious argument: The SED relation ${\tau }_{\ell }\propto \eta$/*T* is violated with the lowest-order rotational relaxation time ${\tau }_{1}$, but is instead satisfied at the higher-order time scale of ${\tau }_{6}$. In contrast, we find that ${D}_{{\rm{t}}}{\tau }_{6}$ deviates from values at high temperatures, similarly to the violation of the translational SE relation ${D}_{{\rm{t}}}\eta$/*T*, while ${D}_{{\rm{t}}}{\tau }_{1}$ superficially satisfies the SED relation.
(iv) We observe the coupling between the translational diffusion constant, *D*_t_, and the lowest rotational relaxation time, ${\tau }_{1}$. We characterize the correlation between large translational and rotational mobilities using from the van Hover correlation function ${G}_{s}(r,\theta ;t)$. Furthermore, we find that ${\tau }_{1}$ exhibits the temperature dependence similar to that of the hydrogen-bond lifetime ${\tau }_{{\rm{HB}}}$, which is consistent with the previously demonstrated result, ${D}_{{\rm{t}}}\propto {{\tau }_{{\rm{HB}}}}^{-1}$ ^63^.
(v) On the contrary, we show that the higher-order rotational relaxation time ${\tau }_{6}$ is analogous with the *α*-relaxation time ${\tau }_{\alpha }$, rather than with ${\tau }_{{\rm{HB}}}$. This time scale is characterized by immobile molecular mobilities showing dynamic heterogeneities, which we investigate using the four-point correlation functions ${\chi }_{{\rm{t}}}(t)$ and ${\chi }_{{\rm{r}}}(t)$. It is also of essential to examine the role of the length scale of dynamic heterogeneities ${\xi }_{4}$ on the violations of the SE/SED relations in supercooled water. The ${\xi }_{4}$ value is conventionally quantified by the wave-number dependence of the four-point correlation functions^76^. This calculation necessitates MD simulations using more substantial large systems, which are currently undertaken.
(vi) In conclusion, in this paper we provide significant and unprecedented insights into the appropriate assessment of SE, Debye, and SED relationships, in doing so clarifying previously awkward and confusing contradictions. Finally, it is worth mentioning the importance of the density dependence on the SE/SED relations in supercooled water. In fact, both $\eta$ and *D*_t_ show anomalous density dependence, particularly at low temperaures^65^. Further investigations along this line are necessary to clarify this issue.

## Methods

### Molecular dynamics simulations

We performed MD simulations of liquid water using the Large-scale Atomic/Molecular Massively Parallel Simulator (LAMMPS)^77^, and used the TIP4P/2005 model to calculate the water molecule potentials^78^. Other MD simulations were also carried out to investigate various properties in supercooled states of this model^65–67,79–86^. The comparison with other rigid non-poralizable models was also made in the recent review^87^. Remark that recent ab initio MD simulations provide a more realistic behavior of the dynamics in supercooled regime^88^. We used a Coulombic cutoff 1 nm. The particle-particle particle-mesh solver was utilized to calculate long-range Coulomb interactions, and the SHAKE algorithm was also used for bond and angle constraints. Periodic boundary conditions were used, and the time step of simulation was 1 fs. First, we employed the *NVT* ensembles for *N* = 1,000 water molecules was employed at various temperatures (*T* = 300, 280, 260, 250, 240, 230, 220, 210, 200, and 190 K) with a fixed mass density of *ρ* = 1 g cm^−3^. The corresponding system size was *L* = 31.04 Å. We conducted the *NVE* ensemble simulations after the equilibration with a sufficient long time at each temperature, The dynamical quantities including, the *α*-relaxation time ${\tau }_{\alpha }$, the translational diffusion constant *D*_t_, and the hydrogen-bond lifetime ${\tau }_{{\rm{HB}}}$, and shear viscosity $\eta$ used in here are reported in a previous study^63^. In this study, we newly calculated time correlation functions for characterzing the rotational diffusion constant *D*_r_, the rotational relaxation time of the $\ell$-th degree Legendre polynomials ${\tau }_{\ell }$. Furthermore, the four-point correlation function was also calculated for characterizing dynamic heterogeneities of rotational molecular motions. The trajectories for the calculations of various quantities were for 10 ns (*T* ≥ 220 K) and 100 ns (*T* ≤ 210 K). We average over five independent simulation runs for the calculations.

### Rotational diffusion constant and rotational relaxation time

We calculated the angular mean-square displacement $\langle {\rm{\Delta }}\varphi {(t)}^{2}\rangle =(1/N)\,{\sum }_{j=1}^{N}\,\langle |{\rm{\Delta }}{\overrightarrow{\varphi }}_{j}(t){|}^{2}\rangle$, following ref.^52^ (see Supplementary Fig. S1(a)). We obtained the angular displacement vector ${\rm{\Delta }}\,{\overrightarrow{\varphi }}_{j}\,(t)$ of the molecule *j* is obtained through the time integration of the angular velocity vector, ${\overrightarrow{\varphi }}_{j}(t)={\int }_{t^{\prime} }^{t^{\prime} +t}\,{\overrightarrow{\omega }}_{j}(t){\rm{d}}t$, where the angular velocity vector ${\overrightarrow{\omega }}_{j}(t)$ of the molecule *j* is given by the cross-product of the normalized polarization vector ${\overrightarrow{e}}_{j}(t)$ as, ${\overrightarrow{\omega }}_{j}(t)={\overrightarrow{e}}_{j}(t)\times {\overrightarrow{e}}_{j}(t+{\rm{\Delta }}t)$/${\rm{\Delta }}t$, with the magnitude $|{\overrightarrow{\omega }}_{j}(t)|={\cos }^{-1}({\overrightarrow{e}}_{j}(t)\cdot {\overrightarrow{e}}_{j}(t+{\rm{\Delta }}t))$. Note that Δ*t* is chosen by a sufficiently small time interval; this was 10 fs in our calculations. We determined the rotational diffusion constant *D*_r_ was determined from the long-time limit of $\langle {\rm{\Delta }}\overrightarrow{\varphi }{(t)}^{2}\rangle$ as ${D}_{{\rm{r}}}={\mathrm{lim}}_{t\to \infty }\,\langle {\rm{\Delta }}\varphi {(t)}^{2}\rangle$/4*t*. Furthermore, we independently calculated the angular velocity time correlation function, ${C}_{{\rm{\Omega }}}(t)=(1/3N)\,{\sum }_{j=1}^{N}\,\langle {\overrightarrow{{\rm{\Omega }}}}_{j}(t)\cdot {\overrightarrow{{\rm{\Omega }}}}_{j}(0)\rangle$, where ${\overrightarrow{{\rm{\Omega }}}}_{j}(t)$ denotes the angular velocity vector of the molecule *j* in the world reference frame following ref.^57^ (see Supplementary Fig. S1(b)). We also used the Green–Kubo formula to obtain *D*_r_ as ${D}_{{\rm{r}}}={\int }_{0}^{\infty }\,{C}_{{\rm{\Omega }}}(t){\rm{d}}t$. We confirmed that the *D*_r_ values obtained from these two methods are consistent at any temperature.

The rotational correlation function ${C}_{\ell }(t)$ is defined by the autocorrelation function of the normalized polarization vector ${\overrightarrow{e}}_{j}(t)$ as ${C}_{\ell }(t)=(1/N)\,{\sum }_{j=1}^{N}\,\langle {P}_{\ell }[{\overrightarrow{e}}_{j}(t)\cdot {\overrightarrow{e}}_{j}(0)]\rangle$, where ${P}_{\ell }[x]$ is the $\ell$-th order Legendre polynomial as a function of *x* (see Supplementary Fig. S2). ${C}_{\ell }(t)$ decays from 1 to 0 as *t* increases. We obtained the $\ell$-th ($\ell =1$, 2, 3, and 6) order rotational relaxation time ${\tau }_{\ell }$ by fitting ${C}_{\ell }(t)$ to the Kohlrausch–Williams–Watts function $A\,\exp \,\{\,-\,{(t/{\tau }_{\ell })}^{{\beta }_{\ell }}\}$.

### Rotational four-point correlation functions

We used the four-point correlation function to elucidate the degree of dynamic heterogeneity in supercooled liquids^76^. The four-point correlation ${\chi }_{{\rm{t}}}(t)$ for translational motions is defined by the variance of the intermediate scattering function ${F}_{s}(k,t)$ as, ${\chi }_{{\rm{t}}}(t)=N\,[\langle {\hat{F}}_{s}{(k,t)}^{2}\rangle -{\langle {\hat{F}}_{s}(k,t)\rangle }^{2}]$, with ${\hat{F}}_{s}(t)=(1/N)\,{\sum }_{j=1}^{N}\,\cos \,[\overrightarrow{k}\cdot {\rm{\Delta }}{\overrightarrow{r}}_{j}(t)]$. We previously calculated ${\chi }_{{\rm{t}}}(t)$ using the wave-number *k* = 3.0 Å^−1^, and quantified the peak time ${\tau }_{{\rm{t}}}$ (note that the same quantity was denoted by ${\tau }_{{\chi }_{4}}$ in ref.^63^). The rotational four-point correlation function ${\chi }_{{\rm{r}}}(t)$ can be analogously defined as ${\chi }_{{\rm{r}}}(t)=N\,[\langle {\hat{C}}_{\ell }{(t)}^{2}\rangle -{\langle {\hat{C}}_{\ell }(t)\rangle }^{2}]$, with ${\hat{C}}_{\ell }(t)=(1/N)\,{\sum }_{j=1}^{N}\,{P}_{\ell }[{\overrightarrow{e}}_{j}(t)\cdot {\overrightarrow{e}}_{j}(0)]$. The peak time of ${\chi }_{{\rm{r}}}(t)$ is represented by ${\tau }_{{\rm{r}}}$.

## Supplementary information

supplementary information

## Data Availability

The data supporting the findings of this study are available from the corresponding authors upon reasonable request.
