# Supplementary material for: Spurious violation of the Stokes–Einstein–Debye relation in supercooled water
Source: Sci Rep. 2019 May 31;9:8118. doi: 10.1038/s41598-019-44517-4 (PMC6544661; doi:10.1038/s41598-019-44517-4)
Supplement: Supplementary file 1 — supplementary information [file 41598_2019_44517_MOESM1_ESM.pdf]

Supplementary Information

Spurious violation of the Stokes–Einstein–Debye relation in supercooled water

Takeshi Kawasaki and Kang Kim

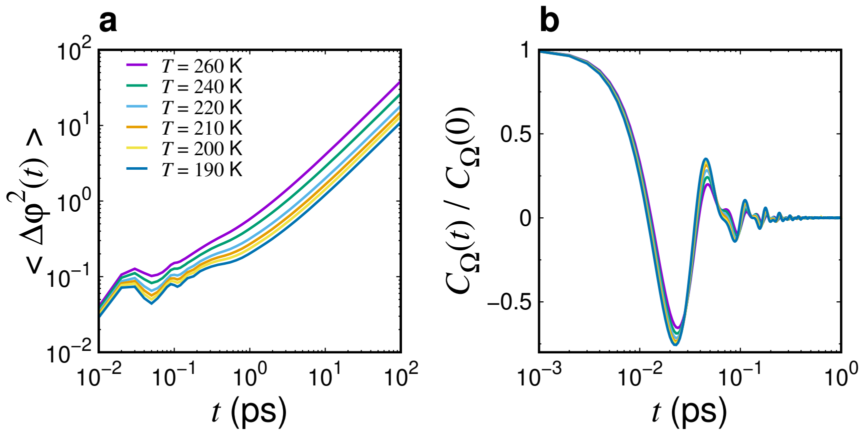

FIG. S1. (a) Mean squared angular displacement $\langle \Delta\varphi(t)^2 \rangle$. (b) Angular velocity correlation function $C_\Omega(t)$.

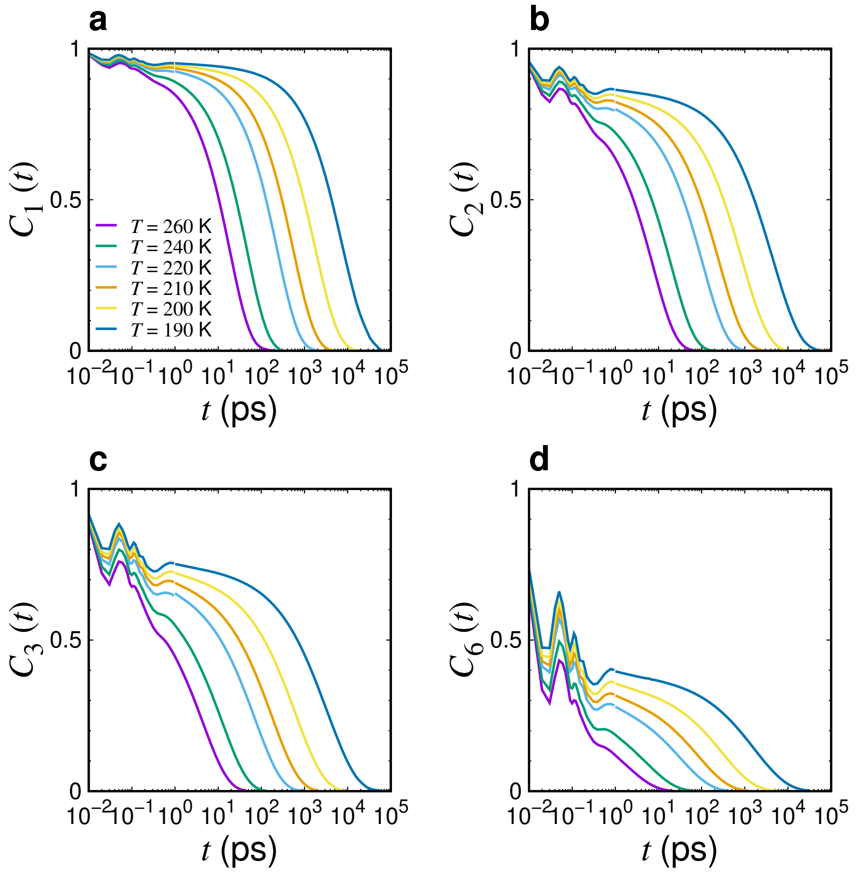

FIG. S2. Rotational correlation function $C_\ell(t)$ for $\ell = 1$ (a), 2 (b), 3 (c), and 6 (d).

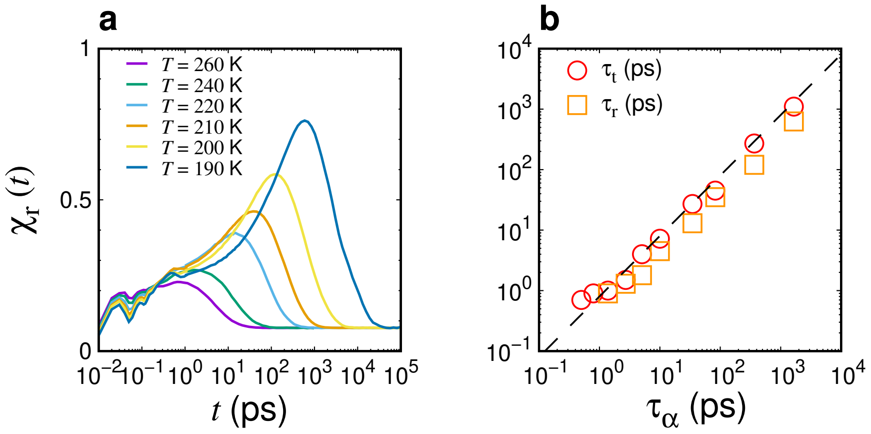

FIG. S3. (a) Rotational four-point correlation functions, $\chi_{\rm r}(t)$ with the order $\ell = 6$. (b) The relationship between $\tau_{\rm t}$ and $\tau_{\rm r}$, and α-relaxation time τ_α, as determined by the intermediate scattering function $F_s(k,t)$, with $k = 3.0$ Å^{-1}.
